# Water Ski Injuries and Chronic Pain in Collegiate Athletes

**DOI:** 10.3390/ijerph18083939

**Published:** 2021-04-09

**Authors:** Hyun Chul Jung, Hanna Straltsova, Michael A. Woodgate, Kyung-Min Kim, Jung-Min Lee, Joon-Hee Lee, Joshua J. Gann

**Affiliations:** 1 Department of Coaching, College of Physical Education, Global Campus, Kyung Hee University, 1732 Deokyoungdaero, Giheung-gu, Yongin-si 17014, Gyeonggi-do, Korea; jhc@khu.ac.kr (H.C.J.); borracho@khu.ac.kr (J.-H.L.); 2 Department of Kinesiology, College of Health Sciences, University of Louisiana at Monroe, 700 University Avenue, Monroe, LA 71209, USA; streltsova.ania@gmail.com (H.S.); woodgama@warhawks.ulm.edu (M.A.W.); 3 Department of Sport Science, College of Sport Science, Sungkyunkwan University, 2066 Seobu-ro, Jangan-gu, Suwon-si 16419, Gyeonggi-do, Korea; km.kim@skku.edu; 4 Department of Physical Education, College of Physical Education, Global Campus, Kyung Hee University, 1732 Deokyoungdaero, Giheung-gu, Yongin-si 17014, Gyeonggi-do, Korea; jungminlee@khu.ac.kr

**Keywords:** water-skiing athletes, injuries, chronic pain

## Abstract

This study examined the rate of injuries and chronic pain in collegiate water-ski athletes as a preliminary study. We also compared the mechanics and cause of injuries by the level of water-skiing experiences. A total number of 96 collegiate water-ski athletes, aged 21.4 ± 2.23 years, participated in the study. An off-line questionnaire was distributed at the collegiate tournaments in the United States. The questionnaire consisted of 20 questions, including demographic information, body region and type of injuries, mechanics and cause of injuries, chronic pain and pain management. A Chi-squared test was used to examine the differences in injury rates by sex and the level of experiences (beginner: <5 years, intermediate: 5–10 years, advanced: <10 years). The significance level was set at ≤0.05. A total of 336 water skiing-related injuries were observed from 96 participants. The ankle/feet, knee, and head/neck regions were the most common body regions injured, representing 26.5, 16.7, and 15.8%, respectively. Female athletes were more likely to have nerve injuries than male athletes (*p* = 0.039). The intermediate athletes were more likely to have trunk (*p* = 0.047) and upper extremity (*p* = 0.042) injuries than beginner athletes, and the beginner athletes had less joint/ligament (*p* = 0.001) and bone injury (*p* = 0.010) compared to the advanced athletes. Torsion/twisting (32.8%) and deceleration (26.9%) were the most common mechanism of injury. Beginner athletes experienced injuries more due to insufficient skill (*p* = 0.03), while the advanced athletes were likely to have more injuries by the loss of control (*p* = 0.01). Collegiate athletes had higher rates of chronic pain in the trunk (42.7%) and skeletal muscle (43.8%), and they participated in stretching/exercise (40.8%) and massage/form rolling (29.6%) to manage their chronic pain. The present study revealed that injury rates in males and females were 49.7% and 50.2%, respectively. Female athletes were more likely to have a nerve injury than male athletes. The mechanics and cause of injuries were different by the level of experiences where different training approaches may be required to minimize the injuries. Additionally, the strength and conditioning program that is systematically designed for core strength is needed to eliminate chronic trunk pain in collegiate water-skiing athletes.

## 1. Introduction

Water skiing is one of the popular extreme sports performed on a water surface. The number of participants in water-skiing activity increased to more than 3.57 million in 2018 in the United States [1]. The International Waterski & Wakeboard Federation (IWWF) is the world governing body for towed water sports and has 91 affiliated national federations. The IWWF’s competitive and recreational towed water sport divisions include Tournament (3-event water skiing), Wakeboard, Barefoot, Show Skiing, Cable Wakeboard, Cable Ski, Ski Racing, and Disabled Skiing [2].

Tournament waterskiing commonly consists of three disciplines: slalom, trick, and jump. Briefly, slalom skiers use only one ski with feet oriented forward, one in front of the other. The skier’s goal is to complete the slalom course, consisting of six turn buoys that the skier navigates in a zigzag pattern, as well as going through entrance and exit gates at the beginning and the end of the pass. Water-ski jumpers use a pair of long skis to ride over a solid ski ramp in an attempt to travel the longest distance. Trick skiing uses smaller, oval-shaped skis and includes surface and wake rotations, flips, and toe tricks performed by attaching one foot to the handle. The harder a trick skill, the more points it values. Thus, skiers execute as many tricks as possible in two passes to obtain as many points as possible, with the trick’s execution assessed by a panel of officials.

Three-event water skiing is widely considered to be the most demanding discipline in towed watersports. Various fitness components such as muscular strength and endurance, dynamic balance, and coordination are required to succeed in waterskiing competition [3]. An intricate blend of techniques with fast decision-making is critical to adjust the movements and respond to alternative situations efficiently [4]. The nature of 3-event water skiing is characterized by high speed, height, a high level of muscular effort, rapid accelerations, and explosive movements. Therefore, the risk of water ski-related injuries might be elevated due to its risk elements and characteristics. The unpredictable nature of falls also makes it possible to incur injury to any area of the body [3]. Disproportional training due to asymmetric posture on skis (one foot forward and the other one backward, in slalom and trick) and repetitive loading leads to muscular imbalance that could predispose to injury as well. Biomechanical analysis and strength tests of water skiers have indicated that muscular imbalances are present in water skiers, such as that upper back strength is two times greater than the chest and abdominal muscle strength [5]. Cold weather and poor equipment may also contribute to the incidence of injuries. Typically, water ski-related injuries differ by the level of experience, where tournament water skiers experience elevated risks of injuries through the elevated speed, the strain on the body, more complex technical elements, and highly competitive situations that encourage an athlete to take additional risks. In contrast, previous research found novice and recreational skiers injured themselves due to a lack of physical fitness and inexperience [6].

Chronic pain is a condition for an unpleasant sensation or pain lasting more than six months, and athletes typically have one or more body regions of chronic pain [7]. Low back and leg pain are prevalent among athletes due to orthopedic surgery, stress fracture, muscular imbalance, inflexibility of the lower extremity, and degenerative disc disease [8,9,10,11]. Tournament water skiing such as slalom and trick is performed with one ski, and the repeated exposure of this movement is associated with the muscle imbalance between left and right of the body where chronic pain could be incurred by muscle imbalance. Insufficient or inappropriate pain management may also increase the severity of chronic pain. Although water-skiing athletes have a potential risk of various chronic pains, lack of information about the body regions of chronic pain and their pain management may limit providing or generating the conditioning program.

To date, little research has investigated water skiing-related injuries. Although some research described water skiing related-injuries, most studies were reported as case studies [12,13,14]. Hostetler and colleagues examined the water skiing-related and wakeboarding-related injuries in the United States from 2001–2003. Hostetler et al. found that the most common injuries treated in emergency departments were lower-limb strains and sprains, as well as trunk injuries [15]. A similar study performed from 2000 to 2007 indicated that hip and lower-extremity injuries were the most common body region injured [16]. However, these data do not reflect overall water skiing-related injuries, as the authors reported cases treated only in the emergency departments. Moreover, there is no literature regarding injury patterns in collegiate water-ski athletes. Collegiate water skiing is popular in the United States; to date, the National Collegiate Water Ski Association includes 78 collegiate teams across the country. However, lack of scientific research on water skiers, especially the mechanics of injuries, may limit the sport’s growth, particularly in the development of safe techniques and training programs. We also believe that the mechanics and the cause of injuries may be different by the level of water-skiing experience. Therefore, this study examined the rate of water skiing-related injuries, chronic pain and pain management in collegiate athletes as a preliminary study. We also compared the mechanics and cause of injuries by the level of water-skiing experience.

## 2. Materials and Methods

### 2.1. Participants

Participants were asked to recall all the water skiing-related injuries and chronic pain that they have had during the collegiate period. Participants included those who had at least 1 year of water-skiing experience, and they had to be an active collegiate water skier as a member of the USA National Collegiate Water Ski Association. A total of 215 collegiate athletes participated in the Fleur de Ski and All-Stars Ski tournaments, but 61 athletes participated in both tournaments; thus, a total of 103 participants from 154 collegiate athletes took part in the survey. A total of 103 participants took part in the survey from two different water-ski competitions (i.e., Fleur de Ski, All-Stars Ski Tournament); however, 7 participants were excluded from the data analysis due to an uncompleted questionnaire (N = 4) and not meeting inclusion criteria (N = 3). Therefore, 96 participants’ (male, N = 46, and female, N = 50) questionnaires were used in this study. Participants’ basic characteristics, as well as water-ski demographic information, are presented in Table 1. The study was approved by the Institutional Review Board of Human Subject Research at the University of Louisiana at Monroe (ULMIRB-838) prior to gathering the data.

### 2.2. Questionnaire

A descriptive survey was implemented to collect injury data. An off-line survey was selected as the data collection method. The questionnaire was distributed at a Regional (Fleur de Ski, Lafayette, LA, U.S.) and a National water-ski tournament (All-Stars, Monroe, LA, U.S.) in the United States. The questionnaire was developed based on the previous studies [17,18]; five experts, including professional water-ski athletes and exercise scientists, assessed the questionnaire, and the first draft was distributed to 10 collegiate water skiers as a pilot survey. The first part of the questionnaire consisted of six questions covering demographic information (e.g., sex, age, career, categories, height, weight). Secondly, participants were asked seven questions to report training information, including training volume, frequency, warm-up, and fitness-training program. The final part of the questionnaire involved type, body region, and mechanism of injuries during water-skiing training or competition and the location the injury occurred. The body region was classified into nine parts, namely head/neck, shoulder, upper back, elbow, wrist/hand, low back, hip/thigh, knee, and ankle/foot based on the Nordic classification system [19]. Injury type was categorized into five types, namely muscle/tendon (strain, tendinitis), joint/ligament (dislocation, subluxation, articular damages, sprain), bone (fracture), skin (abrasion, laceration), and nerve (concussion, other nerve injuries) based on the previous studies [20,21]. The question included four mechanics of injury that can occur during water-skiing training or competition such as compression, torsion or twisting, pulling, and deceleration. Then, participants were asked to select the cause of injuries, including insufficient skill, loss of control, weather condition, low physical fitness, and poor equipment. If the cause of injury fell out of the categories, participants were asked to describe in the text box labeled “other”. In addition, participants were asked to report their chronic pain and pain management. Chronic pain was categorized into four body regions (i.e., head/neck, trunk, upper extremity, and lower extremity) and four pain types (bone, muscle, tendon/ligament, others). Athletes also reported their pain management such as sauna, local heat pack, icing/ice bathing, massage/form rolling, stretching/exercise, and nutritional supplement.

In this study, water skiing-related injury was defined in the questionnaire as “a physical complaint or damage to body tissue during water skiing training or competition that causes medical attention or its consequence for impairments associated with training or competition.” [18]. Chronic pain was defined as “an individual who has an unpleasant sensation or pain lasting more than six months from the water-skiing related-injury” [7].

### 2.3. Statistical Analyses

Statistical analyses were performed using the SPSS software program version 25 (IBM, Chicago, IL, USA). Participants’ basic information (e.g., age, height, body mass, body mass index) and water-ski demographics (e.g., career, training volume on and off-season) were computed with mean and standard deviation. For descriptive analyses including frequency, a proportion were performed to examine basic injury information, including the location and type, chronic pain, and pain management. A Chi-squared test was applied to examine the statistically significant differences of injury type, body region injured, mechanisms, and the cause of injury by sex and the level of water-ski experience. The level of water-ski experience was categorized into three career years (beginner, <5 years; intermediate, 5–10 years; advanced, 10> years). If any significant differences were identified, a post hoc test was applied to identify the individual differences between the levels of experience. Additionally, the Cramer’s V (ϕc) coefficients were calculated to examine the strength of the significance where 0.00–0.10 indicates a low association, while 0.11–0.30, 0.31–0.51, and 0.51–1.00 represent moderate to substantial, substantial to strong, and very strong association, respectively [22]. The significance level was set as ≤0.05.

## 3. Results

### 3.1. Body Region and Type of Injuries in Collegiate Water Ski Athletes

Table 2 shows the number and percentage of injuries by body region and injury type in collegiate water-ski athletes. A total of 336 water skiing-related injuries were observed from 96 participants. Male athletes accounted for 49.7% (*n* = 167) of injuries, and female athletes accounted for 50.3% (*n* = 169) of injuries during the collegiate period. Overall, lower-extremity injuries, including knee (16.7%), ankle and feet (26.5%) injuries represented the largest percentage of water skiing-related injuries. In comparison, upper extremities, including shoulder (5.4%) and elbow (4.8%) injuries, showed the lowest rates of injuries among collegiate athletes. Concerning the type of injuries, the highest rate of injury type was skeletal muscle (33.3%), while nerve (13.7%) injury demonstrated the least common injury type. There were no significant associations between sex and body part injured; however, there was a significant association between sex and nerve injury (χ^2^(1, 46) = 4.727, *p* = 0.039, ϕc = 0.199), where female athletes had more nerve injury than male athletes.

### 3.2. Distribution of Body Part and Type of Injury Within Disciplines

**Figure 1:**
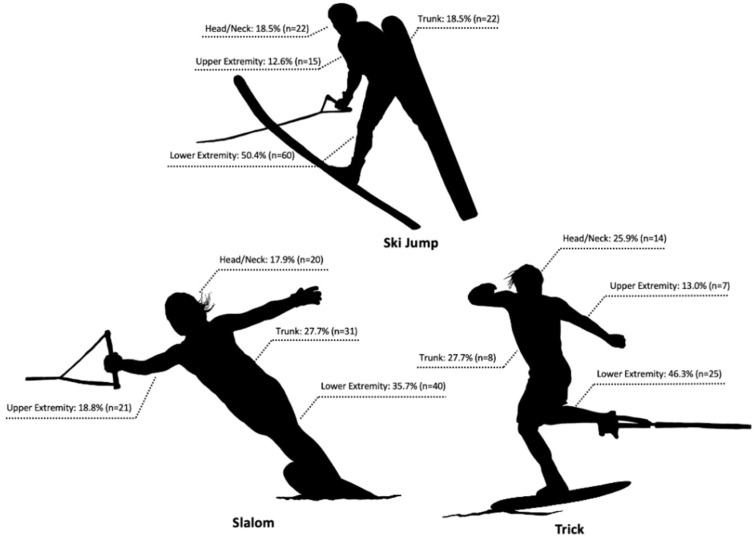
The distribution of body parts injured by 3-event water skiing.

The distribution of water skiing-related injury by disciplines is shown in Figure 1.

### 3.3. Distribution of Type and Location of Injury by the Levels of Water Ski Experiences

In this study, collegiate water-ski athletes are divided into three groups by the level of water-skiing experiences (beginner level, <5 years; intermediate level, 5–10 years; advanced, 10 > years). The advanced athletes were more likely to have a head/neck injury compared to the beginner athletes (χ^2^ (2, 109) = 9.100, *p* = 0.010, ϕc = 0.291). The intermediate athletes had a higher rate of trunk (χ^2^ (2, 113) = 6.118, *p* = 0.047, ϕc = 0.233), and upper extremity (χ^2^ (2, 121) = 6.355, *p* = 0.042, ϕc = 0.229) injuries compared to the beginner athletes, but no significant difference was observed with the advanced athletes. No significant association between lower-extremity injury and the level of water-ski experience was observed. Regarding the injury type, the intermediate and the advanced athletes had a higher rate of joint/ligament (χ^2^ (2, 128) = 13.160, *p* = 0.001, ϕc = 0.329) and bone-related injuries (χ^2^ (2, 113) = 9.123, *p* = 0.010, ϕc = 0.284) compared to the beginner athletes. The relationship between the type and location of the injury and the level of water-skiing experience is shown in Table 3.

### 3.4. Mechanics and Cause of Injury

Table 4 represents the distribution of the mechanics of injury. Overall, torsion/twisting (32.8%) and deceleration (26.9%) were the most common mechanisms of injury. The beginner athletes were less likely to have injuries induced by torsion/twisting than the intermediate and the advanced athletes (χ^2^ (2, 128) = 12.959, *p* = 0.002, ϕc = 0.318). No significant differences in other mechanisms of injury such as compression, pulling, and deceleration were observed by the level of experience.

Regarding the cause of injury, loss of control (46.2%) was the most common cause of injury. The beginner athletes self-reported that they experienced injury more due to insufficient skill compared to the advance athletes (χ^2^ (2, 96) = 7.347, *p* = 0.025, ϕc = 0.277). But the advanced athletes demonstrated that they were likely to have more injury by the loss of control than the beginner-level athletes (χ^2^ (2, 96) = 10.909, *p* = 0.006, ϕc = 0.324). The results of the cause of injury are presented in Table 5.

### 3.5. Chronic Pain and Pain Management

Table 6 shows the results of chronic pain and pain management. Overall, collegiate water-skiing athletes had higher rates of chronic pain in the area of the trunk (42.7%), whereas head/face/neck presented the lowest body region of chronic pain (8.3%). The most common type of chronic pain was skeletal muscle (43.8%). A higher number of athletes participated in stretching and exercise (40.8%) to manage their chronic pain. Massage and form rolling were the second most common treatment method (29.6%), whereas the athletes rarely participated in a sauna (3.4%) or nutritional supplement (4.7%) for chronic pain management.

## 4. Discussion

We investigated the rate of water skiing-related injuries to identify factors such as sex or level of experience that possibly contribute to the injuries in collegiate water-ski athletes. The main findings of this study were that injury rates in males and females were 49.7% and 50.2%, respectively. Female athletes were more likely to have a nerve injury than male athletes. Beginner athletes experienced injuries more due to insufficient skill, while the advanced athletes were likely to have more injuries by the loss of control. Collegiate athletes self-reported higher rates of chronic pain in the trunk, and they participated in stretching/exercise and massage/form rolling to eliminate their chronic pain.

### 4.1. Body Region and Type of Injuries in Collegiate Water Ski Athletes

In the present study, the incidence rates of injury between males and females were similar, representing 49.7% and 50.2%, respectively. A consistent result was observed in the previous study where the injury rates were 60.2% in males, and 64.3% in female water-ski athletes [6]. No significant sports injuries between males and females were also observed among NCAA Division III athletes [23]. In contrast, Hostetler et al. also demonstrated that the rate of injury in males accounted for 72.2% [15]. These inconsistent results with the present study may be associated with the number of female participants. While the present study was undertaken with athletes that had about 0.9 sex ratio (male to a female), other studies included recreational participants that possibly reflect the lower female participants [6], thus it is assumed that different sex ratios between studies can affect the result of sex effect on injury. Although no significant associations between sex and body region injured were observed in the present study, the interesting finding was that female athletes had a higher rate of nerve injury in the area of head/neck, and upper and lower back compared to male athletes. It suggests that more medical attention may be required to address concussions and other nerve injuries among female collegiate athletes during training or competition.

Overall, ankle/feet (26.5%), knee (16.7%), and head/neck (15.8%) regions were the most common body region injured. Previous studies support our result that low-extremity injury is the most common site of body region injured [15,16]. Typically, water skiers experience excessive load at the ankle to maintain the balance on the fluid water surface. These loads are transferred through the ankle with skiers wearing stiff, high bindings. Sharp turns and rapid accelerations may increase the risk of ligament injuries on the knee in the slalom event. Interestingly, the head/neck injury was the third most common injury among collegiate water-ski athletes. Baker et al. also reported that head/neck injury was the second most common water skiing-related injury (24.6%) [16]. However, water-ski athletes had a lower risk of head/neck injury than other towed water sports, such as wakeboard and tubing [15,16]. There is a common similarity between towed water sports where athletes are being towed along the surface of the water by a motorboat, but different techniques or rules between sports resulted differently in the body part injured. Dependent on the discipline and standard, water skiers experience elevated speed (slalom and jump) compared to wakeboarders. Whilst the advanced slalom water skier turns quickly at high speed, a wakeboarder will ride much slower, performing rotations and inverts across the wake, similar to a trick skier. In particular, incomplete flipping and under-rotations in wakeboarding could increase the risk of head/neck injury through a whiplash motion. Water skiers also have the inherent risk of head/neck injury during a rapid sharp turn or deceleration, which should be considered. The inherent risk of head injury in the jump event is managed with athletes being required to wear helmets, often covering their head and face. Wakeboarders are also encouraged to wear helmets; however, participants in slalom and trick do not wear helmets.

While the intermediate athletes were more likely to have trunk and upper-extremity injuries, the advanced athletes had more head/neck injuries compared to the beginner athletes. These may be due to the duration of experience of the athlete, where beginner athletes generally had been participating for a shorter duration than a collegiate athlete. Previous studies reported there were no significant correlations between the number of injuries and the competitive level [6,24]. The authors pointed out that even though the cause of injuries was different between the competitive levels, it did not affect the overall rate of injuries. This is a paradox where the advanced athletes could experience fewer injuries due to better performance, but performing at a higher speed creates more risk of injury, which resulted in no difference between the competitive levels [6]. In the present study, muscle/tendon injury was the most common type of injury (33.3%), but there was no significant association with sex and level of experience. Other studies also demonstrated that sprain and strain were the major type of water skiing-related injury, representing 64.6% [6], 36% [15]. The intermediate and the advanced athletes had more joint/ligament and bone injuries than the beginner athletes. This suggests that the risk of joint/ligament and bone injury becomes greater as the water-ski experience increases, possibly due to the elevated technical requirements and speed. Intermediate or advanced level athletes perform more technical maneuvers, at higher speeds. In attempting to gain that extra buoy in short-line slalom, jump that extra few feet or fit that extra trick into a pass, elevated forces are present with smaller margins of error that can result in more severe injuries such as bone fracture and ligament injuries.

### 4.2. Body Region Injured by Discipline

In the present study, a higher number of injuries were observed in the slalom (39.3%) and ski jump (41.8%) than the trick (18.9%). Our findings are supported by previous studies where slalom and ski jump had higher rates of injuries than trick [24]. Loughlin reported that the injuries that occurred during slalom and ski jump accounted for 76.8% and 23.3%, respectively, while no injury was reported in the trick [6]. It is reasonable that slalom and ski jump events required high speed to maximize performance, and performing a variety of techniques at high speed is the major cause of water skiing-related injuries [25]. For instance, the maximal speed of the boat in the slalom event is 34.2 mph for female and 36 mph for male adults, but the velocity a skier can experience from turning the buoy, to learning across the wakes to the next buoy, will often see the skier reach speeds above 50 mph during short-line slalom skiing [5]. In the same context, a male adult water-ski jumper has a maximum boat speed of 35 mph; however, an experienced water-ski jumper can reach speeds over 70 mph before hitting the ramp. Previous research indicated the different causes of injuries between the events, which possibly contribute to the results of body region injured [26].

Regardless of all three water-ski events, the lower-extremity injury (43.9%) was observed as the most common injury. While water-ski jump had the highest percentage of lower-extremity injury (50.4%), the rates of trunk injury were the highest in the slalom. A similar result has found that the most common body region injured was the lower extremity in the ski jump event, and trunk injury was the most common injury in the slalom event [6]. When a slalom skier attempts to turn buoys sharply, at high speed, maintaining an aligned body position from the end of the turn is required to move across the course to the next buoy efficiently. However, slalom skiing is a very technical event, influenced by many factors such as water temperature, water texture, wind speed, wind direction, boat manufacturer, and many more. Thus, slalom skiers may make a mistake, such as losing their aligned body position, failing to remain balanced, experience uneven weight distribution, or encounter rough water and cause a skier to fall, potentially creating a “whip-like effect” on the trunk [15]. Furthermore, ski jumpers can make similar mistakes, finding themselves imbalanced or out of position, and hit the jump ramp in an undesirable position that can cause a skier to experience a rough landing from the jump, which could increase the risk of injury at the lower extremity. Trick skiers also experience a higher number of lower-extremity injuries that commonly occur during toe-hold tricks due to the single-leg loading and increased torsion/twisting motion.

### 4.3. Mechanism and Cause of Injury

There were no significant sex effects on the mechanism and the cause of injury in the present study. Overall, collegiate athletes experienced higher rates of injuries by torsion/twisting (32.8%) and deceleration (26.9%), where the ski catches the water while the body is still moving. The beginner athletes were less likely to have injuries by torsion/twisting than the intermediate and the advanced athletes.

Whilst the size of slalom and trick skis depends more on skiers’ height and weight than their ability, more advanced slalom ski athletes often have more aggressively shaped and less forgiving skis that are more suitable to high-performance than the wider, more forgiving intermediate-level skis. Advanced jump skiers require longer skis, whereas novice and intermediate level jumpers often use smaller skis. Typically, ligamentous injuries occur from a ski catching the water, whilst one’s mass continues to travel causing a torsion/twisting effect that places excessive strain on the ligamentous tissues. The increased forces involved with performing rapid rotational movements (tricks) and skiing at higher speeds (slalom and jump) may be associated with higher rates of joint/ligament injuries compared to beginner athletes.

Overall, the major cause of injuries was the loss of control (46.1%). This result was expected and substantiates previous research where falling was the most common injury among water skiers [6,24]. However, the beginner athletes reported that they were more likely to have injuries by insufficient skill, while the advanced athletes had higher rates of injury by the loss of control than the beginner athletes. Our result suggests that different strategies need to be applied based on the level of experience. Notably, beginner athletes need a physical conditioning program that is specially designed to improve water skiing-related fitness such as dynamic balance, core strength, and stability.

### 4.4. Chronic Pain and Pain Management

In the present study, collegiate water-ski athletes had higher rates of chronic pain in the trunk and skeletal muscle. Typically, low back pain is a prevalent symptom among the athletic population [8]. Previous research supports our result that chronic low-back pain was the most common symptom in water skiers [27]. Various factors such as muscular imbalance, inflexibility of the lower extremity, and degenerative disc disease have been associated with low-back pain [8,10,11]. We assumed that chronic lower-back pain in slalom skiers is due to repeated exposure to excessive forces and poor form. Additionally, one-ski events such as slalom and tricks promote a muscular imbalance in the hip and leg. This muscular imbalance is known to be associated with low-back pain occurrence in collegiate athletes [11]. Collegiate water-ski athletes commonly participated in stretching/exercise (40.8%) and massage/form rolling (29.6%) to manage their chronic pain. However, most of the athletes participated in self-selected activities, such as resistance exercise, yoga, and cycling. We believe that more systemized conditioning programs need to be developed as a part of the chronic trunk-pain prevention program for water-ski athletes.

### 4.5. Strength and Limitation of the Study

This study has some strengths and limitations that need to be considered when interpreting the data. First, this is the first observational study, mainly focusing on collegiate water-ski athletes, but a small sample size may not represent the overall injury rates of collegiate water skiers. Additionally, the self-reported questionnaire would have common limitations such as a recall bias that needs to be considered in the study. However, this study provides new insight into injury location, type, mechanism, and cause of injury for developing future studies. Second, athletes reported their injury experienced in a retrospective manner, and relying on the memory would create room for error [28]. The severity of the injury was not examined in the present study. Finally, we examined the injury rates during the collegiate period. Therefore, participants’ college experience as an athlete would be different.

### 4.6. Practical Application

Water skiing is one of the popular extreme sports among collegiate athletes, but the risk of injury is potentially high due to the nature of its characteristics and various environmental factors. This preliminary study provides the overall information of injury rate, mechanics, and cause of injuries by the level of water-skiing experiences. Based on our results, coaches and athletes would be able to design the specialized training program or safety guidelines that need to be considered by experience level. Particularly, dynamic balance training may help advanced athletes minimize the injury by loss of control. Collegiate water-skiing athletes typically manage their chronic pain by themselves with various methods. Coaches and athletic trainers may develop a systematically designed program to manage athletes’ chronic pain. Future studies may be recommended to examine the severity of the injury and the longitudinal observation from the freshmen to final years to systemically track the prevalence of injuries and the rate of dropout from sports due to injuries during collegiate periods.

## 5. Conclusions

The present study revealed that injury rates in males and females were 49.7% and 50.2%, respectively. Female athletes were more likely to have a nerve injury than male athletes. Beginner athletes experienced injuries more due to insufficient skill, while the advanced athletes were likely to have more injuries by loss of control; thus, different training approaches may be considered to minimize the injuries. Additionally, a strength and conditioning program that is systematically designed for core strength is needed to eliminate chronic trunk pain in collegiate water-skiing athletes.

## Figures and Tables

**Table 1:** Participants’ basic characteristics and water-ski demographics.

Athletes’ Physique Information	Total (N = 96)
Female participant (%)	52.1
Age (years)	21.4 ± 2.23
Body mass (kg)	69.7 ± 10.72
Height (cm)	172.6 ± 9.33
Body mass index (kg/m^2^)	23.3 ± 2.63
**Water Ski Demographics**	
Career (years)	11.8 ± 6.29
Training in the off-season (h/wk)	8.6 ± 5.20
Training in in-season (h/wk)	6.1 ± 3.94

**Table 2:** Location and type of injuries in collegiate water-ski athletes.

	Muscle/Tendon	Joint/Ligament	Bone	Skin	Nerve
Head/Neck 53(30) (15.8%)	8(4)	0(0)	4(3)	11(5)	30(18)
Shoulder 18(6) (5.4%)	5(2)	9(2)	2(1)	1(0)	1(1)
Upperback 25(14) (7.4%)	11(5)	3(1)	5(3)	4(3)	2(2)
Elbow 16(8) (4.8%)	1(1)	7(2)	3(1)	3(3)	2(1)
Wrist/Hand 30(15) (8.9%)	6(1)	5(4)	11(4)	7(5)	1(1)
Low back 28(16) (8.3%)	17(8)	2(1)	2(1)	1(1)	6(5)
Hip/Thigh 21(12) (6.3%)	11(5)	8(6)	1(0)	0(0)	1(1)
Knees 56(33) (16.7%)	18(10)	25(14)	2(2)	9(6)	2(1)
Ankle/Feet 89(35) (26.5%)	35(18)	18(6)	19(5)	16(6)	1(0)
Total 336(169) (100%)	112(54) (33.3%)	77(36) (22.9%)	49(20) (14.6%)	52(29) (15.5%)	46(30) * (13.7%)

Note. A number in the parentheses represents the frequency of injury among female athletes. * indicates a significant association between sex and nerve injury where females had more injury than male athletes.

**Table 3:** Relationship between type and location of injury and years of skiing.

	Total	<5 Years (N = 21)	5–10 Years (N = 17)	10 Years < (N = 58)	$\chi^{2}$ (*p*-Value)
**Body Region**					
Head/Neck	53 (100%)	4 ^a^ (7.5%)	10 ^ab^ (18.9%)	39 ^b^ (73.6%)	9.20 (0.01)
Trunk	53 (100%)	7 ^a^ (13.2%)	15 ^b^ (23.8%)	31 ^ab^ (58.5%)	6.12 (0.05)
Upper extremity	64 (100%)	8 ^a^ (12.5%)	19 ^b^ (29.7%)	37 ^ab^ (57.8%)	6.36 (0.04)
Lower extremity	166 (100%)	26 (15.7%)	33 (19.9%)	107 (64.5%)	3.65 (.16)
Total	336				
**Injury Type**					
Muscle/Tendon	112 (100%)	26 (23.2%)	27 (24.1%)	59 (52.7%)	0.47 (.79)
Joint/Ligament	77 (100%)	5 ^a^ (6.5%)	19 ^b^ (24.7%)	53 ^b^ (68.8%)	13.85 (0.001)
Bone	49 (100%)	3 ^a^ (6.1%)	12 ^b^ (24.5%)	34 ^b^ (69.4%)	9.12 (0.01)
Skin	52 (100%)	6 (27.3%)	10 (45.5%)	36 (50.0%)	3.52 (0.17)
Nerve	46 (100%)	5 (23.8%)	9 (45.0%)	32 (47.8%)	3.81 (0.15)
Total	336				

Note. The same alphabet indicates no significant difference between the level of experience.

**Table 4:** The distribution of mechanics of injury.

	Compression	Torsion/Twisting *	Pull	Deceleration
Head/Neck 40(25) (14.8%)	13(8)	6(2)	7(5)	14(10)
Trunk 48(27) (17.7%)	10(6)	15(8)	15(7)	8(6)
Upper Extremity 43(20) (15.9%)	8(4)	15(6)	15(8)	5(2)
Low Extremity 140(71) (51.6%)	21(11)	53(27)	20(12)	46(21)
Total 271(144) (100%)	52(29) (19.2%)	89(43) (32.8%)	57(33) (21.0%)	73(39) (26.9%)

Note. A number in the parentheses represents the frequency of injury among female athletes. * indicates a significant association with the level of experience. The intermediate and advanced athletes were more likely to have torsion and twisting injuries than the beginner athletes (χ^2^ (2, 128) = 12.959, *p* = 0.002, ϕc = 0.318).

**Table 5:** The distribution of the cause of injury by the level of ski experiences.

	Total	<5 Years (N = 21)	5–10 Years (N = 17)	10 Years < (N = 58)	$\chi^{2}$ (*p*-Value)
**Cause of Injury**					
Insufficient Skill	24 (100%)	10 ^a^ (41.7)	3 ^ab^ (12.5)	11 ^b^ (45.8)	7.35 (0.03)
Loss of Control	60 (100%)	7 ^a^ (11.7)	11 ^ab^ (18.3)	42 ^b^ (70.0)	10.09 (0.01)
Weather Condition	5 (100%)	0 (0%)	2 (40.0)	3 (40.0)	2.63 (0.27)
Low Physical Fitness	12 (100%)	2 (16.7)	3 (25.0)	7 (58.3)	0.59 (0.74)
Poor Equipment	13 (100%)	1 (7.7)	3 (23.1)	9 (69.2)	1.82 (0.40)
Others	16 (100%)	2 (12.5%)	2 (12.5%)	12 (75.0)	1.74 (0.42)
Total	130				

Note. The same alphabet indicates no significant difference between the level of experience.

**Table 6:** Distribution of chronic pain and pain management.

		Bone	Muscle	Tendon /Ligament	Others
**Body Region**					
Head/Neck	8 (8.3%)	2	4	1	1
Trunk	41 (42.7%)	6	26	7	2
Upper extremity	14 (14.6%)	4	4	6	0
Lower extremity	33 (34.4%)	9	8	14	2
Total	96 (100%)	21 (21.9%)	42 (43.8%)	28 (29.2%)	5 (5.2%)
**Pain Management**					
Sauna	8 (3.4%)				
Local heat pack	18 (7.7%)				
Icing/ice bathing	32 (13.7%)				
Massage/Form rolling	69 (29.6%)				
Stretching/Exercise	95 (40.8%)				
**Nutritional Supplement**	11 (4.7%)				
Total	233 (100%)				

## Data Availability

The data presented in this study are available on request from the corresponding author. The data are not publicly available due to privacy.
